# Draft Genome Sequences of Two Desiccation-Tolerant Strains, Bradyrhizobium japonicum TXVA and TXEA, Isolated from the Root Nodules of Soybean Grown in Texas

**DOI:** 10.1128/mra.00467-22

**Published:** 2022-08-02

**Authors:** Christian Peterson, Sarbjeet Niraula, Dylan Parks, Woo-Suk Chang

**Affiliations:** a Department of Biology, University of Texas at Arlington, Arlington, Texas, USA; University of Arizona

## Abstract

Two Bradyrhizobium japonicum strains, TXVA and TXEA, were isolated for their desiccation tolerance and symbiotic performance with soybean as biofertilizers. Their genomes were sequenced and annotated using the Department of Energy Joint Genome Institute annotation pipeline. Sequencing yielded chromosomes of 9,193,770 and 9,339,455 bp for TXVA and TXEA, respectively.

## ANNOUNCEMENT

Bradyrhizobium japonicum, a nitrogen-fixing symbiont, has been widely applied as an inoculant in soybean (Glycine max) fields (1). However, drought presents a huge impediment to the application of this inoculant due to inhibition of the symbiosis caused by poor survival of the symbiont under desiccation stress (2). Thus, the primary aim of this research was to bioprospect for desiccation-tolerant *Bradyrhizobium* strains. Soybean root nodules were collected in 30% glycerol across drought-prone agricultural fields in Texas. The nodules were crushed into 500 μL of sterile water and serially diluted. Each dilution (100 μL) was spread onto yeast mannitol agar with Congo red. Using the filter disk desiccation assay (3), we were able to isolate two desiccation-tolerant B. japonicum strains, TXVA and TXEA, from the root nodules of soybeans grown in Victoria and Lubbock counties in Texas, respectively. Strains TXVA and TXEA showed 87.1% ± 0.61% and 93.9% ± 0.63% survivability, while the wild-type strain B. japonicum USDA110 revealed 80.00% ± 0.98% survivability under the desiccation conditions (i.e., 27% relative humidity) (4). The two strains are Gram-negative, rod-shaped, nitrogen-fixing bacteria and have produced increases in the number of root nodules and seed yield in plants treated with them compared to the wild type (4). Here, we report the genome sequences of B. japonicum TXVA and TXEA. Single colonies isolated for both strains were cultured in arabinose-gluconate (AG) medium at 30°C with shaking at 200 rpm, followed by genomic DNA (gDNA) extraction using the GeneJET genomic DNA purification kit (Thermo Fisher Scientific). The concentration (>100 ng/μL) and purity (260/280 ratio, 1.8) of the gDNA were determined using a NanoDrop spectrophotometer (Thermo Fisher Scientific). Libraries were prepared using the NEBNext Ultra II DNA kit (New England BioLabs) for Illumina according to the manufacturer’s instructions, and sequencing was performed at the University of Texas at Austin Genomic Sequencing and Analysis Facility (GSAF) on an Illumina MiSeq 3 (paired-end [PE] 2 × 300-bp read format) platform. Sequencing of TXVA and TXEA produced 6,950,108 and 6,968,744 reads with approximately 227× and 224× coverage, respectively. The raw reads were quality filtered, and trace adapter sequences were subsequently removed using BBDuk, a program developed at the DOE Joint Genome Institute (DOE-JGI), with the parameters k = 23, mink = 11, hdist = 1, tpe tbo qtrim = rl, trimq = 15, ftl = 5, ftr = 294, ftm = 5, maq = 15, and minlen = 100. The filtered reads were quality checked using FastQC (5). Default parameters were used for all software, unless otherwise specified. Assembly of the filtered reads was performed using SPAdes 3.15.3 (6), and the assembly quality was assessed using QUAST 5.0.2 (7). The assembled genome sequence for TXVA contained 70 contigs (≥1,000 bp) totaling ~9.2 Mbp, with an *N*_50_ value of 621,423 bp. The assembled genome sequence for TXEA contained 71 contigs (≥1,000 bp) totaling ~9.3 Mbp, with an *N*_50_ value of 528,185 bp.

Functional annotation and gene prediction were performed using the DOE-JGI Microbial Genome Annotation Pipeline (8). JGI’s Integrated Microbial Genomes (IMG) system (9) revealed that the assembled genome sequence of strain TXVA consists of 9,193,770 bp, with 8,980 protein coding genes (72.95% with predicted functions), 4 rRNA genes, 64 tRNA genes, and an average G+C content of 63.67%. The complete genome of strain TXEA consists of 9,339,455 bp, with 9,158 protein coding genes (72.99% with predicted functions), 3 rRNA genes, 63 tRNA genes, and an average G+C content of 63.64%.

### Data availability.

The whole-genome sequencing projects of B. japonicum TXVA and TXEA have been deposited at NCBI GenBank under the accession numbers JAKRCG000000000 and JAKRCH00000000, respectively. The raw sequences have been deposited in the NCBI SRA database under the accession numbers SRR17988758 and SRR17988757 for strains TXVA and TXEA, respectively.
